# Comparison of morphometry and ventricular function of healthy and smoking young people

**DOI:** 10.1186/s12872-020-01372-w

**Published:** 2020-02-06

**Authors:** Ana Natália Ribeiro Batista, Thais Garcia, Estefânia Aparecida Thomé Franco, Paula Schmidt Azevedo, Mauricio Fregonesi Barbosa, Leonardo Antonio Mamede Zornoff, Marcos Ferreira Minicucci, Sergio Alberto Rupp de Paiva, José William Zucchi, Irma de Godoy, Suzana Erico Tanni

**Affiliations:** Department of Clinical Medicine of the Universidade Estadual Paulista (UNESP, Paulista State University), at Botucatu School of Medicine, Botucatu, São Paulo, Brazil

**Keywords:** Cardiovascular magnetic resonance, Smoking, Heart, Ventricular function

## Abstract

**Background:**

Tobacco smoke is one of the most significant risk factors for cardiovascular diseases and damages in the myocardial tissue directly. Cardiac magnetic resonance (CMR) has been used and is a promising tool to evaluate morphometry and cardiac function in humans. The objective of this study was to evaluate associations of smoking with morphometry and cardiac function by CMR technique in young adult smokers.

**Methods:**

Altogether, 49 volunteers (22 smokers and 27 non-smokers) were included in the study. The comparisons between groups were performed by multiple linear regression adjusting for body mass index and gender.

**Results:**

In the morphometric and functional evaluation of the left ventricle, we observed statistical significant lower values of end-diastolic volume (EDV) (*p* = 0.02), ejection volume (EV) (*p* = 0.001) and indexed ejection volume (IEV) (*p* = 0.007) in smokers when compared to no-smoker group. Right ventricle showed statistical significant lower values of EDV (*p* = < 0.001), end-systolic volume (*p* = 0.01), EV (*p* = < 0.001), IEV (*p* = 0.001), indexed end-diastolic volume (*p* = 0.001) and major axis (*p* = 0.01) in smokers when compared to non-smokers group.

**Conclusions:**

There is a strongly association of smoking in young adult and cardiac function decline, even adjusted by cofounders, which compromises the proper functioning of the heart. Evidence confirms that smoking can directly influence the cardiac function, even without atherosclerosis or other chronic comorbidities, associated with increased risk of cardiovascular diseases.

## Background

Smoking is a public health problem worldwide and is the leading cause of preventable death. Approximately 6 million people die every year because of tobacco-related diseases. The prediction is that ten million deaths occur per year in the world in 2030. In Brazil, approximately 200,000 deaths per year are estimated due to smoking [1, 2]. Tobacco smoke is one of the most significant risk factors for cardiovascular diseases and damages myocardial tissue directly [1]. Long-term smoking is associated with considerable metabolic and morphological changes in the heart muscle that can be characterized as smoking cardiomyopathy, including significantly modifications in right and left chambers functions, resulting in diastolic or systolic dysfunctions [3, 4]. However, experimental studies show that exposure to tobacco smoke, which has over 4700 chemical substances, increases the incidence of cardiovascular diseases [4–7]. Another experimental study showed that the toxic effects of tobacco smoke was associated with eccentric hypertrophy, regardless of hemodynamic effects [6]. In addition, the findings were correlated with apoptosis, hypertrophy and myocardial dysfunction [8–10]. A clinical study by Nadruz et al. [11], which evaluated more than 4500 elderly healthy individuals through transthoracic echocardiography showed that active smoking and cumulative exposure to cigarettes were associated with subtle changes in the structure and function of the left ventricle, but not evaluated the right chambers’. Besides echocardiography, cardiac magnetic resonance (CMR) has been used and is a promising tool to evaluate morphometry and cardiac function in humans [12]. However, we identified no data in the literature that assess cardiac function of smoking patients without cardiovascular diseases by CMR. Thus, the main objective of this study was to evaluate associations of smoking with morphometry and cardiac function through the CMR technique in young adult smokers.

## Methods

### Study population

We evaluated 84 individuals of both genders, smokers (active smokers with at least smoking history ≥10 packs-year) and control (never smokers) from an university city close to the center-west region of São Paulo State. In the smoking group, we included subjects with > 18 years old and we excluded those with acute infection diagnosis or in use of medicines in the past 4 weeks, pregnancy, claustrophobia, or any chronic disease such as coronary insufficiency, cardiac insufficiency, systemic arterial hypertension (SAH), diabetes *mellitus*, dyslipidemia, respiratory, hepatic, renal, psychiatric diseases, cancer and changes in the laboratory tests [hemogram, fasting glycemia, C-reactive protein, triglycerides, total cholesterol or high-density lipoprotein (HDL)] or in the pulmonary function test. We included 22 subjects in the smoking group and 19 were excluded due to the following reasons: one for pregnancy, four for change in laboratory analysis, seven for abnormal lung function and seven withdrew the informed consent form (ICF).

In the control group, we included subjects aged > 18 and those who presented a history of never use of any tobacco product. The exclusion criteria were the same from the smoker group. Altogether, 27 subjects were included in the control group and 16 were excluded due to the following reasons: one for SAH, one for previous bariatric surgery, one for endometriosis, one for claustrophobia, one for diabetes, one did not sign the ICF, five for laboratory analysis changes and five for pulmonary function changes.

All participants included in this study signed the informed consent form approved by the Research Ethics Committee of the Clinical Hospital of Botucatu Medical School.

### Data source

All subjects of the research were evaluated through clinical history and complete physical examination. Smoking history (packs-years) and current smoking state were investigated and complemented by assessing intensity of nicotine dependence (Fargeström Test) [13] and confirmation of active smoking was accomplished through carbon monoxide (CO) measurement in the exhaled air through a standardized technique with specific equipment (Micro+ Smokerlyzer, Bedfont, England, UK) [14, 15]. Physical activity was assessed by International Physical Activity Questionnaire (IPAQ) short version [16].

Spirometry was performed (only to assess inclusion and exclusion criteria) in a computerized portable system with pulmonary function (Ferraris KOKO, Louisville, CO, USA), according to American Thoracic Society criteria [17]. We measured forced vital capacity (FVC) in liters (L) and forced expiratory volume in the first second (FEV1) in liters (L) and calculated ratio of the two measures (FEV1/FVC). Measures were obtained before and 20 min after administration of 400 mcg of salbutamol dosed as bronchodilator medication. FVC and FEV1 values were also expressed in percentage of the reference values [18].

All participants of the research underwent CMR examination, which were carried out in the 3-T MR device (Magnetom Verio, Siemens AG, Health care Sector, Erlangen, Germany) according to the study protocol. The localizers were obtained through image cut sequences of the heart to the programming of sequences of posterior images. Images on cine-MRI in short and long axes of the left ventricle (LV) using the Steady-State Free Precession sequence were used for calculations of ventricular volumes and functions. The T1 mapping (that allows measuring fibrosis areas in the myocardial tissue) was performed by the Modified Look-Locker Inversion Recovery – MOLLI sequence with motion correction, not available for purchase (Work-in-progress – WIP). T1 mapping were obtained through diastole, in the middle segment of the short axis of the left ventricle and through images of four cameras. The T2 mapping (that allows measuring areas of edema and inflammation in the myocardial tissue) was obtained in the middle segment of the left ventricle.

After acquisition of these images, gadolinium contrast was injected (Gadolinium DTPA – 0.15 mmol/kg) and new images of late enhancement were obtained after 15 min using the phase-sensitive inversion-recovery – PSIR sequence in the short and long axes of the LV and also the T1 maps at the same anatomical plans.

### Analysis of images

Ventricular function, volumes and mass of the LV were calculated through the Ventricular Function Argus software (Siemens AG, Healthcare Sector). All volumes and ventricular mass were indexed to the body surface area [19]. Using standardized segmentation of the left ventricle we split the T1 mapping into sixteen myocardial segments for T1 time measurements independently [20]. The Apex (segment 17) was not analyzed due to the impossibility of avoiding partial volume effect in this segment. Regions of interest (ROIs) were drawn in the pre-contrast image and then copied to the post-contrast images. Calculation of the extracellular volume (ECV) was carried out manually, using the T1 measures before and 15 min after administration of the intravenous contrast [12]. The T2 measurement was performed with the “ROIs” positioned in the interventricular septum to avoid that any increases in the native T1 result from edema.

### Statistical analysis

We used the Statistical Package for Social Sciences (SPSS) 17.0 (Inc, Chicago, IL, USA). Descriptive analysis of the results was performed, and the data are presented in absolute numbers, percentage and/or in mean and standard deviation and median and 25/75 percentile. For the comparison between two groups (smoker and control), the Student’s t test was used for parametric distribution variables and the Mann-Whitney test for the nonparametric distribution variables. We used multiple linear regression analysis to compare control vs smokers adjusted by gender and body mass index (BMI). We used multiple linear regression analysis with robust standard errors when the normality was not assumed. All models considered control group as a comparator. Therefore, the coefficient needs to be interpreted as an estimated mean difference between smoking group and control group, such as, when the coefficient is negative, the smoking group presented lower values compared to control group. We considered a 5% significance level.

## Results

General characteristics of the 49 subjects are presented on Table 1. The smokers showed an average smoking history of 17.7 ± 6.7 packs-years and 32% of these showed a high degree of nicotine dependence. The smokers had significantly smaller stature and higher body mass index and as expected, the CO mean in exhaled air was higher in the smoking group compared to the control group. IPAQ did not show statistical significance between groups.

**Table 1:** Characteristics of groups

Variables	Non-smoker (*n* = 27)	Smoker (*n* = 22)	*P* value
Male (%)	59.3	50.0	0.71
Age (years)	34.0 (30.0–39.0)	34.5 (30.8–39.3)	0.96
Weight (Kg)	75.6 ± 12.6	74.5 ± 15.6	0.78
Height (m)	1.73 ± 0.11	1.66 ± 0.10	0.02
BMI, (Kg/m^2^)	24.9 (22.8–26.5)	26.8 (24.6–29.1)	0.03
CO (ppm)	2.00 (1.00–3.00)	8.50 (4.75–14.3)	< 0.001
SB (pack years)	–	17.68 ± 6.72	–
IPAQ	270.0 (0.0–540.0)	90.0 (0.0–255.0)	0.11

*BMI* Body Mass Index, *CO* carbon monoxide, *SB*smoking burden, *IPAQ* International Physical Activity Questionnaire. Data are expressed as mean ± standard deviation or median with 25 and 75 percentiles

When we compared morphometric and functional variables of the LV between both groups, we observed significantly lower values of ejection volume (EV) and the indexed ejection volume (IEV) (Table 2). When the model was adjusted for gender and BMI using multiple linear regression analysis to compare both groups, we observed statistical significant lower values of EV, IEV and end-diastolic volume (EDV) in smoker group compared to control group (Table 3).

**Table 2:** Functional and morphometric evaluation of the LV by cardiac magnetic resonance imaging between control group and smokers

Variables	Non-smoker (*n* = 27)	Smoker (*n* = 22)	*P* value
EF (%)	63.7 ± 5.47	61.5 ± 4.58	0.14
EDV (mL)	144 ± 30.9	130 ± 24.4	0.09
ESV (mL)	52.9 ± 17.2	50.3 ± 12.5	0.56
EV (mL)	90.9 ± 16.6	79.5 ± 15.9	0.02
LVM (g)	117 ± 28.4	116 ± 30.0	0.95
IEDV (mL/m^2^)	75.6 ± 10.4	71.0 ± 8.73	0.10
IESV (mL/m^2^)	27.6 ± 6.75	27.5 ± 5.51	0.94
IEV (mL/m^2^)	47.9 ± 5.92	43.5 ± 5.32	0.009
ILVM (g/m^2^)	61.6 ± 10.6	63.4 ± 10.4	0.54
IVS (mm)	10.0 (9.00–11.0)	9.00 (8.00–10.2)	0.20
LVPW (mm)	8.70 ± 1.81	8.45 ± 2.01	0.65
LVEDD (mm)	52.9 ± 5.33	51.3 ± 4.14	0.26
LVESD (mm)	34.1 ± 3.92	34.0 ± 3.98	0.96

*EF* ejection fraction, *EDV* end-diastolic volume, *ESV* end-systolic volume, *EV* ejection volume, *LVM* left ventricular mass, *IEDV* indexed end-diastolic volume, *IESV* indexed end-systolic volume, *IEV* indexed ejection volume, *ILVM* indexed left ventricular mass, *IVS* interventricular septum, *LVPW* left ventricular posterior wall, *LVEDD* left ventricular end-diastolic diameter, *LVESD* left ventricular end-systolic diameter. The data are expressed as mean ± standard deviation (parametric distribution) or median 25 and 75 percentiles (non-parametric distribution)

**Table 3:** Comparison between groups (smokers and controls) by multiple linear regression of morphometric and functional variables of LV adjusted by gender and body mass index

Variables	Coeficiente (95%CI)	*P* value
EF (%)	−2.59 (−5.62;0.44)	0.09
EDV (mL)	−14.7 (−27.1;-2.37)	0.02
ESV (mL)	−2.35 (−10.1; 5.34)	0.54
EV (mL)	−12.4 (− 19.3; −5.37)	0.001
LVM (g)	−1.05 (− 11.3; 9.12)	0.83
IEDV (mL/m^2^)	−4.25 (−9.48; 0.98)	0.11
IESV (mL/m^2^)	0.29 (−3.21; 3.79)	0.87
IEV (mL/m^2^)	−4.44 (−7.63;-1.26)	0.007
ILVM (g/m^2^)	2.15 (−2.31; 6.62)	0.34
IVS (mm)^a^	−0.42 (−1.44; 0.61)	0.41
LVPW (mm)	−0.35 (−1.31; 0.60)	0.46
LVEDD (mm)	−1.59 (−4.36; 1.18)	0.25
LVESD (mm)	−0.07 (−2.21; 2.07)	0.95

*EF* ejection fraction, *EDV* end-diastolic volume, *ESV* end-systolic volume, *EV* ejection volume, *LVM* left ventricular mass, *IEDV* indexed end-diastolic volume, *IESV* indexed end-systolic volume, *IEV* indexed ejection volume, *ILVM* indexed left ventricular mass, *IVS* interventricular septum, *LVPW* left ventricular posterior wall, *LVEDD* left ventricular end-diastolic diameter, *LVESD* left ventricular end-systolic diameter. Multiple linear regression reference group: controls

^a^Multiple linear regression with robust standard errors. The coefficient needs to be interpreted as an estimated mean difference between smoking group and control group, such as, when the coefficient is negative, the smoking group presented lower values compared to control group

Comparing morphometric and functional variables of the right ventricle (RV) of smokers and controls, we observed statistical significantly lower values of EV, IEV, EDV, indexed end-diastolic volume (IEDV) and major axis in smokers (Table 4). Adjusting the model by BMI and gender using multiple linear regression analysis we identified EV, IEV, EDV, IEDV, end-systolic volume (ESV) and major axis statistical significantly lower values in smoker group when compared to control group (Table 5).

**Table 4:** Functional and morphometric evaluation of the RV by cardiac magnetic resonance imaging between control group and smokers

Variables	Non-smoker (*n* = 27)	Smoker (*n* = 22)	*P* value
EF (%)	63.7 ± 5.47	61.5 ± 4.58	0.14
EDV (mL)	140 ± 36.5	114 ± 29.7	0.01
ESV (mL)	60.2 ± 21.5	49.5 ± 15.8	0.05
EV (mL)	79.7 ± 18.5	64.4 ± 16.0	0.004
IEDV (mL/m^2^)	73.4 ± 14.1	61.8 ± 10.6	0.003
IESV (mL/m^2^)	31.3 ± 8.83	27.0 ± 6.64	0.06
IEV (mL/m^2^)	42.1 ± 8.28	35.0 ± 5.53	0.001
Major axis (mm)	81.0 ± 10.6	72.5 ± 9.04	0.005
Minor axis (mm)	45.0 (40.0–53.0)	42.0 (38.7–45.2)	0.30

*EF* ejection fraction, *EDV* end-diastolic volume, *ESV* end-systolic volume, *EV* ejection volume, *IEDV* indexed end-diastolic volume, *IESV* indexed end-systolic volume, *IEV* indexed ejection volume. Data are expressed as mean ± standard deviation (parametric distribution) or median with 25 and 75 percentiles (non-parametric distribution)

**Table 5:** Comparison between groups (smokers and controls) by multiple linear regression of morphometric and functional variables of RV adjusted by gender and body mass index

Variables	Coeficiente (95%CI)	*P* value
EF (%)	−1.37 (− 4.62; 1.88)	0.40
EDV (mL)	−25.8 (− 39.7; − 11.1)	< 0.001
ESV (mL)	−9.7 (− 17.5; − 1.85)	0.01
EV (mL)	− 16.1 (− 24.4; − 7.87)	< 0.001
IEDV (mL/m^2^)	− 10.9 (− 17.1; − 4.70)	0.001
IESV (mL/m^2^)	−3.51 (− 7.13; 0.11)	0.06
IEV (mL/m^2^)	−7.28 (− 11.4; − 3.16)	0.001
Major axis (mm)	−6.96 (− 12.2; − 1.68)	0.01
Minor axis (mm)	−1.66 (− 5.07; 1.76)	0.33

*EF* ejection fraction, *EDV* end-diastolic volume, *ESV* end-systolic volume, *EV* ejection volume, *IEDV* indexed end-diastolic volume, *IESV* indexed end-systolic volume, *IEV* indexed ejection volume. Multiple linear regression reference group: controls. The coefficient needs to be interpreted as an estimated mean difference between smoking group and control group, such as, when the coefficient is negative, the smoking group presented lower values compared to control group

The left atrium and the aorta variables did not show statistically significant alterations between the groups (Table 6). None of subjects of this study presented alterations on maps T1 or T2.

**Table 6:** Comparison between groups (smokers and controls) by multiple linear regression of left atrium and aortic variables adjusted by gender and body mass index

Variables	Coeficiente (95%CI)	*P* value
LA (mm)	0.78 (−2.38; 3.95)	0.62
RA (mm)	− 0.30 (−2.77; 2.17)	0.81
descending aorta (mm)	−0.65 (2.86; 1.55)	0.55
ascending aorta (mm)	−0.31 (−1.65–1.04)	0.65

*LA* left atriumm, *RA* aortic root. Multiple linear regression reference group: controls. The coefficient needs to be interpreted as an estimated mean difference between smoking group and control group, such as, when the coefficient is negative, the smoking group presented lower values compared to control group

## Discussion

The main finding was to identify reduced left and right cardiac function in active smoking in young smokers without cardiovascular diseases. Our study identified lower values of EDV, EV and IEV in the LV and EDV, ESV, EV, IEV, IEDV and major axis in the RV.

Comparing our results with longitudinal study Multi-Ethnic-Study of Atherosclerosis (MESA) with more than six thousand individuals with different cardiovascular risk factor with 14% current smokers and 47% never smokers, presented that current smokers were younger than never-smokers and the association of smoking status and vascular dynamics and function was similar with our results, an unexpected lack of association between smoking and vascular distensibility evaluated by carotid ultrasound and aortic MRI [21]. In the same cohort, but analyzing the relationship of left ventricular mass and geometry to incident cardiovascular events showed that patient who had coronary heart disease were current or former smokers and increase values of LV mass/ LV volume ratio was predictive of incident of coronary heart disease [22].

Under normal conditions, pulmonary vascular compliance is suitable and pulmonary vascular resistance is reactive enough to accept emphatically higher quantities of pulmonary blood flow with minimal increases in pulmonary blood pressure [23]. The pulmonary hypertension was not technically evaluated, however, we can assume our hypothesis to justify the biggest compromising of the right chamber when comparing with the left chamber is that the toxic smoke of the cigarette causes more and/or earlier damage to pulmonary flow leading to vasoconstriction in the pulmonary arteries due to presence of oxidative stress and infiltration of the inflammatory process compared to the systemic flow [3]. This continuous cyclical process would be associated with elevation of pulmonary artery pressure and consequently bigger effort from the right ventricle, which would result in reduced value of volume compared to the control group. This hypothesis is consistent with the experimental study on mice exposed to tobacco smoke, which showed that the right ventricle systolic function presented reduction despite the echocardiogram showed the left ventricular function as normal. Besides, in this study, the authors did not identify changes in mitochondrial respiration between the fibers of the left and right chambers that could justify changes only in the right cardiac function but showed reduced endothelial vasodilatation of the aortic ring [24]. In the same direction, results of an experimental study in pigs corroborate our hypothesis. Both groups of pigs, the one exposed to tobacco smoke and the one exposed to chronic hypoxia, showed that these both injuries produced similar increases in elevation of pulmonary artery pressure and in the right ventricle and that the combination of both agents had a synergetic effect on these changes. In addition, distensibility of the aorta was lower than distensibility of the pulmonary arteries and thickness of the pulmonary arteries presented thickening, which demonstrates the direct effect of tobacco smoke on reactivity and mechanical properties of large pulmonary arteries and in morphological characteristics of small intrapulmonary vessels [25]. However, the literature shows directly influence of smoking on vascular stiffness and arterial age of increased values of serum lipids, continuous activation of sympathetic nervous system and the rise of blood pressure. These chronic modifications associated to smoking leads to an increased risk of cardiovascular disease [ 26].

Our studied population was asymptomatic smoking young adults; however, they already presented reduced value of volume parameter. Changes identified through the CMR in young smokers can be a useful tool for early diagnosis of cardiac changes caused by smoking, which can help behavioral modification of individuals that leads them to quit smoking. However, we cannot say that the reduced value of cardiac functional identified in our sample are definitive. Aggressive cyclic events of smoking and induction for definitive changes may be slow before morphometry and heart function present clinical changes as seen in the experimental studies. At this stage, the heart muscle is still functional and seems completely fine, but in myocardium, there are rising signs of degeneration. Cellular hypertrophy and fibrosis are factors that increase risk of cardiac arrhythmias, heart attack and even sudden death. Still regarding experimental studies with similar results, study by Minicucci et al. [27], which evaluated rats exposed and not exposed to tobacco smoke for 2 months, showed that rats exposed to tobacco smoke presented cardiac remodeling characterized by atrium and LV increase and with decreased systolic function. In addition, they identified mechanisms associated with systolic function worsening, with myocyte hypertrophy, change in energetic metabolism due to increase in lactate dehydrogenase and reduction in citrate synthase, and also increase in oxidative stress (increase in hydroperoxide of lipid proteins and reduction in the superoxide disproportionation). However, the present study did not evaluate the possible inflammatory mechanisms or oxidative stress that can demonstrate similarity with the experimental studies.

Thus, other studies in young smokers that quit smoking are required for proof of the benefit of smoking cessation. Our study did not identify muscular fibrosis. However, CMR images are the only non-invasive method capable of detecting both modifications simultaneously; currently, the interstitial fibrosis is only found through the cardiac tissue biopsy, an invasive and difficult to be applied technique. Through obtention of multiple images of the myocardial fibers, which forms a movie of the heart functioning, the CMR examination allows to calculate size of cells of the heart muscle and quantity of fibrosis. On the other hand, such examination is still considered expensive and its clinical practice application is focused on identification of cardiovascular pathologies with clinical manifestations.

The high accuracy and the smallest variability presented by MRI measurements are of great importance in clinical research, being appropriate to perform serial measurements over time. According to this, the evaluation of early modification of cardiac function in asymptomatic smokers can be compared to experimental studies and it is a tool to evaluate the influence of stopping smoking in early stages of structural cardiac changes.

Therefore, evidence confirms that smoking is strongly related to occurrence of cardiovascular events and cardiac function decline, which compromises the proper functioning of the heart.

This study has some limitations that need to be addressed. First, other studies are necessary to confirm our results, because of the size of the sample is small and consisting only of young smokers. Second, we did not confirm the cotinine levels to assess the smoking impact in possible involved mechanisms. Third, this was a cross-sectional study that cannot affirm the causality. There was no follow-up of smokers to evaluate evolution of cardiac functions and their long-term outcomes. In addition, our study did not evaluate inflammatory or oxidative stress factors associated with the CR mechanism.

## Conclusion

There is a strongly association of smoking in young adult and cardiac function decline, even adjusted by cofounders, which compromises the proper functioning of the heart. Evidence confirms that smoking can directly influence the cardiac function, even without atherosclerosis or other chronic comorbidities, associated with increased risk of cardiovascular diseases.

## Data Availability

The datasets used and/or analysed during the current study are available from the corresponding author on reasonable request.

## Abbreviations

BMI: Body mass index
CMR: Cardiac magnetic resonance
CO: Carbon monoxide
ECV: Extracellular volume
EDV: End-diastolic volume
ESV: End-systolic volume
EV: Ejection volume
FCV: Forced vital capacity
FEV1: Forced expiratory volume in the first second
HDL: High-density lipoprotein
ICF: Informed consent form
IEDV: Indexed end-diastolic volume
IEV: Indexed ejection volume
IPAQ: International physical activity questionnaire
L: Liters
LV: Left ventricle
ROIs: Regions of interest
RV: Right ventricle
SAH: Systemic arterial hypertension
